# Mortality Certified as Diabetic Ketoacidosis Among Brazilian Children and Adolescents, 2015-2023: A National Time-Series Study of Trend and Cause-of-Death Coding Specificity

**DOI:** 10.7759/cureus.117509

**Published:** 2026-10-05

**Authors:** Flaubert Sena de Medeiros Filho, Heli Clovis de Medeiros Neto, Isaac Matheus Bezerra Gurgel

**Affiliations:** 1 Medicine, Universidade Potiguar, Natal, BRA; 2 Surgery, Universidade Federal do Rio Grande do Norte, Natal, BRA

**Keywords:** adolescent health, brazil, cause of death coding, datasus, diabetic ketoacidosis, icd-10, mortality trends, pediatric mortality, time series analysis, type 1 diabetes

## Abstract

Background

Diabetic ketoacidosis (DKA) is a preventable metabolic emergency and an important cause of acute diabetes-related morbidity and death in youth. Contemporary national data describing fatal pediatric DKA in Brazil are scarce. We examined temporal trends and demographic and regional variation in DKA mortality among Brazilian residents aged 0-19 years.

Methods

We conducted a population-based time-series study using final, publicly available records from the Brazilian Mortality Information System for 2015-2023. Deaths were included when the underlying cause was DKA (International Classification of Diseases, 10th Revision codes E10.1, E11.1, E12.1, E13.1, or E14.1) and age at death was 0-19 years. Official age-, sex-, region-, and year-specific population estimates were used as denominators. Rates per one million person-years and exact Poisson 95% confidence intervals (CIs) were calculated. Temporal change was estimated using quasi-Poisson regression with log population as an offset.

Results

We identified 709 DKA deaths during 541.98 million person-years (pooled rate, 1.308 per million person-years). Annual deaths increased from 48 in 2015 to a peak of 109 in 2021 and were 85 in 2023; rates were 0.767 (95% CI: 0.565-1.017) in 2015, 1.846 in 2021, and 1.473 (95% CI: 1.177-1.822) in 2023 per million. The average annual increase was 7.58% (95% CI: 3.66%-11.65%; p < 0.001), but a segmented model joining at 2021 fitted better: +12.52% per year (95% CI: 9.22%-15.91%) to 2021 and -10.12% per year (95% CI: -17.80% to -1.73%) thereafter. All diabetes deaths (E10-E14) increased more slowly (2.59%; 95% CI: 0.22%-5.01%), while the share coded with a ketoacidosis fourth character rose from 35.0% to 58.2%; decomposition attributed 88.9% of the 2015-to-2023 rise in DKA deaths to this coding change. The crude 2020-2023 versus 2015-2019 rate ratio (RR) was 1.40 (95% CI: 1.11-1.75), but 1.03 (95% CI: 0.68-1.55) after adjustment for the secular trend. Rates were highest at age 15-19 years (2.654 per million person-years) and lowest at age 5-9 years (0.487; RR: 5.45; 95% CI: 3.71-8.01). Females had higher mortality than males (RR: 1.38; 95% CI: 1.08-1.76). The Northeast had the highest regional rate and the South the lowest (RR: 1.74; 95% CI: 1.20-2.52). Most deaths occurred in hospitals (90.6%), and 59.2% were coded as DKA in unspecified diabetes (E14.1).

Conclusions

Mortality certified as DKA among Brazilian children and adolescents rose to a peak in 2021 and then declined, with marked age, sex, and regional variation. Most of the measured rise is consistent with more specific fourth-character coding rather than a proportional increase in diabetes deaths, and the 2020-2023 elevation is explained by the pre-existing trend rather than by a pandemic step change. These data cannot establish whether fatal ketoacidosis itself became more frequent; the high proportion of unspecified diabetes coding (E14.1) limits etiologic interpretation and is itself a surveillance finding.

## Introduction

Diabetic ketoacidosis (DKA) is an acute metabolic emergency characterized by hyperglycemia, ketosis, and metabolic acidosis. It can occur at the clinical onset of diabetes or in established disease after insulin omission, interruption of access, intercurrent illness, psychosocial stress, or device failure. The International Society for Pediatric and Adolescent Diabetes emphasizes prompt recognition, careful fluid and insulin therapy, electrolyte monitoring, and vigilance for cerebral injury [1,2]. Although death is uncommon where timely specialized care is consistently available, DKA remains an important cause of acute diabetes-related death in children and adolescents.

Presentation with DKA is not distributed randomly. International studies associate it with younger age, delayed diagnosis, minority or migrant status, and limited access to care, while adolescents with established diabetes face distinct risks related to self-management and transition of care [3,4]. DKA at diagnosis also varies widely between countries and has shown upward trends in several registries [5]. Public-awareness campaigns can reduce DKA at diabetes onset when they reach families, schools, and frontline clinicians [6].

Brazil has a universal health system but persistent social and geographic differences in access to continuous diabetes care. A multicenter Brazilian survey found that 42.3% of patients with type 1 diabetes had been diagnosed during DKA, with variation by economic status, age, and region [7].

A Southern Brazilian referral-center study similarly found that most included children had DKA at type 1 diabetes diagnosis [8]. National research through 2015 evaluated broad diabetes hospitalization and mortality but did not isolate DKA deaths using the four-character International Classification of Diseases, 10th Revision (ICD-10) subcategories [9]. Recent work on diabetes admissions has also emphasized regional patterns and rising burden in younger age groups [10].

The COVID-19 period intensified concern regarding delayed diabetes diagnosis and disrupted chronic care. Meta-analyses and multinational registries reported increased pediatric DKA at diabetes onset during 2020-2021 [11-13], but evidence on fatal DKA in Brazil remains limited.

A national mortality series can address this gap, but only to a limited extent. Vital statistics record what was written and coded on the death certificate, not what was biochemically verified. A study of national files therefore measures mortality certified as DKA, and any observed change may reflect the frequency of the fatal event, the specificity with which clinicians and coders assign a fourth character, or both. We treat that ambiguity as something to be measured rather than acknowledged, and state our aims accordingly.

Objectives

The primary objective is to estimate the temporal trend, from 2015 through 2023, in mortality with DKA recorded as the underlying cause of death (ICD-10 codes E10.1, E11.1, E12.1, E13.1, and E14.1) among Brazilian residents aged 0-19 years, using the general resident population of that age as the denominator.

Secondary objectives include the following: to characterize the shape of that time path rather than assume a constant annual change, by testing for departure from log-linearity and by fitting a segmented model; to quantify how much of any measured change is attributable to increasing specificity of cause-of-death coding, by comparing the DKA series with all pediatric deaths attributed to diabetes (E10-E14) and decomposing the change into a coding-share component and a diabetes-mortality-volume component; to compare mortality in 2020-2023 with 2015-2019, estimating this contrast both crude and conditional on the secular trend, in order to determine whether the pandemic period represents a step change distinct from the underlying trajectory; to describe variation by age group, sex, and macroregion as population rates and to describe the distribution of race/color, place of death, and specific diabetes code among certified deaths; to assess the robustness of the primary estimate to the case definition (including ICD-10 E1x.0, diabetic coma; multiple-cause mentions; and the pre-investigation underlying-cause code), to age bounds, and to exclusion of the pandemic years.

Race/color, place of death, and specific diabetes code are described as distributions of certified deaths only. No population rates are estimated for those variables, because compatible joint denominators are not published for this series.

## Materials and methods

Study design and population

We conducted a retrospective population-based ecological time-series study of deaths among Brazilian residents aged 0-19 years from January 1, 2015, to December 31, 2023. The individual death record was the unit for descriptive analyses; calendar-year and demographic-stratum aggregates were the units for rate analyses. The study used final national files and did not sample hospitals or patients.

Data sources

Nonidentified death records were obtained from the Brazilian Mortality Information System (Sistema de Informações sobre Mortalidade (SIM)) open-data files maintained by the Ministry of Health Department of Health Informatics (DATASUS). We downloaded the national resident-death (DOBR) file for each year from the official repository, following the preprocessing conventions of the Microdatasus workflow [14]. Because the DOBR family is already residence-based, no additional residence filter was required, and nonresidents are not present. SIM consolidates standardized death-certificate information nationwide; a record-linkage evaluation estimated national completeness at approximately 98% in 2016, while noting residual subnational underregistration [15].

Annual population denominators by age, sex, and geographic region were obtained from the DATASUS TabNet table "Population Estimates by Municipality, Age and Sex, 2000-2025" (definition file popsvs2024br.def), coordinated by the Interagency Health Information Network demographic group using the Brazilian Institute of Geography and Statistics data. We used the 2024 (SVS/SVSA) vintage, which incorporates the revision following the 2022 Census. Denominators were taken as published by TabNet in five-year age groups (0-4, 5-9, 10-14, 15-19); single-year ages were not used, and municipal values were not summed by the authors. The national 0-19 denominator is the sum of the four pediatric age groups and reconciles, for every year, with the sums of the sex-specific and region-specific pediatric tables. Data extraction occurred on September 5, 2026. The extraction date is documented because source files may be revised.

Variables and case definition

The underlying cause of death was taken from the SIM variable CAUSABAS, which carries the final code after SIM investigation and recoding. The pre-investigation code (CAUSABAS_O) was retained for one sensitivity analysis. Codes are stored without the decimal point, so E10.1 appears as E101.

An eligible case for the primary analysis met both criteria: (1) age at death from birth through 19 completed years and (2) CAUSABAS equal to E101, E111, E121, E131, or E141. Multiple-cause analyses used the certificate line fields LINHAA, LINHAB, LINHAC, LINHAD, and LINHAII, in which each code is four characters and asterisk-prefixed. The field ATESTADO was not used because it stores some codes as three characters and therefore cannot distinguish a fourth character of .1 from .0.

The SIM age field (IDADE) encodes a unit and a quantity: units of minutes, hours, days, and months were assigned zero completed years, and year-based values were decoded directly. Ages were grouped as 0-4, 5-9, 10-14, and 15-19 years. Sex (SEXO), race/color (RACACOR), municipality of residence (CODMUNRES), and place of death (LOCOCOR) were extracted. The first two digits of CODMUNRES give the state, which was mapped to the five official macroregions. LOCOCOR was recoded as hospital (1), other health facility (2), home (3), public place (4), and other (5 and 6, the latter introduced in the 2021 layout), with remaining values as unknown. RACACOR was recoded as White (1), Black (2), Asian (3), Brown/mixed (4), and Indigenous (5), with remaining values as unknown. The complete mapping is given in the data dictionary accompanying the analysis archive.

Fetal deaths (TIPOBITO = 1) were excluded. Records whose age could not be decoded were excluded; the number of such records carrying a diabetes or a DKA underlying cause is reported, so that potential loss of eligible cases can be judged. Ill-defined underlying causes (ICD-10 Chapter XVIII, R00-R99) were not excluded, and no cause redistribution was performed; their frequency at ages 0-19 is reported as an indicator of source-data quality. No record was excluded for missing sex, race/color, or residence.

In ICD-10, E1x.1 denotes diabetes with ketoacidosis without mention of coma, whereas E1x.0 denotes diabetic coma, with or without ketoacidosis [16]. The primary definition therefore excludes an adjacent and clinically severe presentation, and we address this directly in a sensitivity analysis rather than only in the limitations.

Statistical analysis

Rates were expressed per one million person-years. Exact (Garwood) Poisson 95% confidence intervals (CIs) were computed for descriptive stratum rates as χ²(0.025, 2d)/2 and χ²(0.975, 2(d+1))/2, divided by person-years. Because these intervals ignore extra-Poisson variation between years, every interval used for a between-year comparison was additionally scaled by the Pearson dispersion φ estimated from the corresponding model, as rate × exp(±1.96 × √(φ/d)). Both interval types are reported in Table 1.

Temporal change was modeled by generalized linear regression with a Poisson distribution, log link, and the natural logarithm of the population as an offset, fitted by quasi-likelihood with the covariance matrix scaled by the Pearson dispersion. Calendar year was entered as a continuous variable centered at 2019. The exponentiated year coefficient minus one is reported as the annual percent change (APC).

Departure from a single log-linear slope was assessed in three ways: a Poisson deviance goodness-of-fit test of the linear model; addition of a quadratic year term, tested by a quasi-likelihood F test; and a one-knot segmented (joinpoint) model, with the knot selected by minimum deviance over the candidate years 2017-2021 and the slope change tested by the coefficient of the linear spline term. Nested quasi-likelihood models were compared by F test, since deviance-based likelihood ratio tests are not valid under quasi-likelihood. Because the series contains nine points, the joinpoint location is descriptive and was not subjected to a permutation procedure of the kind used for longer cancer series [17].

The 2020-2023 versus 2015-2019 contrast was estimated in two ways: crude, from a model containing only a period indicator, and conditional on the secular trend, from a model containing both the period indicator and continuous year. The second estimate answers whether the pandemic period departs from the trajectory the series was already on.

Coding specificity was analyzed by comparing the DKA series with all pediatric deaths attributed to diabetes (any fourth character of E10-E14), both obtained from the same microdata extraction, so that the study runs on a single pipeline. The change in DKA deaths between 2015 and 2023 was decomposed multiplicatively as D = N × p, where N is all diabetes deaths, and p is the share coded with a ketoacidosis fourth character; on the log scale, the two components sum exactly to the total. The trend in p was estimated by a quasi-Poisson model of DKA counts with log(all diabetes deaths) as the offset.

Directly age-standardized rates used the pooled 2015-2023 person-year distribution across the four age groups as the standard, with gamma-method CIs [18]. An age-adjusted trend was additionally estimated from year-by-age-group cells with age group as a factor.

Pooled stratum rate ratios (RRs) for age, sex, and region were estimated from quasi-Poisson models fitted to year-by-stratum cells, so that their CIs carry the same dispersion scaling as the trend estimates; reference categories were ages 5-9 years, male sex, and the South region. No adjustment was made for multiple descriptive comparisons, and p-values were not used to rank subgroups.

Analyses used Python 3.12.13 with pandas 3.0.5, NumPy 2.5.3, SciPy 1.18.1, and statsmodels 0.15.0. Tests were two-sided with p<0.05 as the statistical threshold. Integrity checks reconciled age-, sex-, and region-specific denominators with annual national totals, and reconciled the microdata count of all pediatric diabetes deaths against the public TabNet interface.

Ethics

Only non-identified, publicly accessible secondary data were used, without contact with individuals: mortality records from the Mortality Information System (SIM) of the Brazilian Ministry of Health, distributed as open data through DATASUS, and population denominators from the DATASUS population-estimates table. The authors had no access to identifiable records. All records concern deceased persons, and every reported result is an aggregate count or rate. Under Article 1, sole paragraph, items II, III, and V of Brazilian National Health Council Resolution 510/2016, public-domain and aggregate nonidentifiable data research is not registered or evaluated by the Comitê de Ética em Pesquisa (CEP)/Comissão Nacional de Ética em Pesquisa (CONEP) system [19]. Informed consent was not applicable.

## Results

Source data, eligible cases, and data quality

The nine annual files contained 12,952,481 death records. After exclusion of fetal deaths and records with undecodable age, 587,369 deaths at ages 0-19 years remained. Of these, 1,466 had diabetes (E10-E14) as the underlying cause and 709 met the primary DKA definition. A further 53 deaths had E1x.0 (diabetic coma) as the underlying cause. DKA was mentioned somewhere on the certificate in 988 deaths, that is, in 279 deaths beyond those in which it was the underlying cause.

Among the 709 primary cases, sex and municipality of residence were complete, and race/color was unknown in 26 (3.7%). Across all ages, six deaths with a DKA underlying cause had an age field that could not be decoded and were therefore excluded; these are the maximum number of eligible cases that missing age could have removed. Ill-defined underlying causes (R00-R99) accounted for 20,249 of the 587,369 pediatric deaths (3.4%), a possible source of under-ascertainment that applies to all years.

The microdata count of all pediatric diabetes deaths agreed exactly with the public TabNet interface in every year (1,466 in total, annual difference of zero), confirming that the single extraction pipeline reproduces the published national figures.

Annual mortality and the shape of the trend

Among Brazilian residents aged 0-19 years, 709 deaths had DKA recorded as the underlying cause during 541,982,641 person-years, for a pooled rate of 1.308 per million person-years. Annual deaths rose from 48 in 2015 to a maximum of 109 in 2021 and were 85 in each of 2022 and 2023. The corresponding rates rose from 0.767 in 2015 to 1.846 in 2021 and were 1.456 and 1.473 in 2022 and 2023 (Table 1, Figure 1).

**Table 1:** Annual mortality certified as diabetic ketoacidosis among Brazilian residents aged 0-19 years, 2015-2023 CI: confidence interval Rates are per one million person-years. Exact intervals are Garwood Poisson intervals. Dispersion-scaled intervals multiply the log-rate variance by the Pearson dispersion of the trend model (1.67) and are the intervals appropriate for comparing years. Fitted values come from the single log-linear model and from the segmented model with a join at 2021

Year	Deaths	Population aged 0-19	Rate per million	Exact 95% CI	Dispersion-scaled 95% CI	Fitted, single slope	Fitted, two segments
2015	48	62,585,161	0.767	0.565-1.017	0.532-1.105	0.964	0.863
2016	62	62,031,611	0.999	0.766-1.281	0.725-1.378	1.037	0.971
2017	75	61,415,667	1.221	0.961-1.531	0.912-1.636	1.116	1.092
2018	76	60,843,576	1.249	0.984-1.563	0.934-1.670	1.200	1.229
2019	82	60,283,590	1.360	1.082-1.688	1.029-1.799	1.291	1.383
2020	87	59,681,764	1.458	1.168-1.798	1.111-1.912	1.389	1.556
2021	109	59,058,644	1.846	1.515-2.226	1.448-2.352	1.495	1.750
2022	85	58,386,723	1.456	1.163-1.800	1.106-1.916	1.608	1.573
2023	85	57,695,905	1.473	1.177-1.822	1.120-1.938	1.730	1.414

A single log-linear slope gave an average annual change of 7.58% (95% CI: 3.66%-11.65%; p < 0.001; Pearson dispersion: 1.67). That summary describes the series poorly. Adding a quadratic year term improved fit (F = 10.64; df: 1, 6; p = 0.02), with a negative quadratic coefficient indicating downward curvature. A one-knot segmented model fitted best with the join at 2021 (lowest deviance among candidate knots 2017-2021), giving +12.52% per year (95% CI: 9.22%-15.91%) from 2015 to 2021 and -10.12% per year (95% CI: -17.80% to -1.73%) from 2021 to 2023, with a significant change of slope (p < 0.001). A change of slope was detected at every candidate knot, so the departure from linearity does not depend on the knot chosen. The omnibus Poisson deviance goodness-of-fit test of the linear model did not reach significance (deviance 11.64 on 7 df; p = 0.11), which is expected for a directed departure in a nine-point series and does not contradict the two directed tests.

The practical consequence is visible at the end of the series: the single-slope model predicts 1.73 per million for 2023 against an observed 1.473 (Figure 1). Statements of the form "mortality increased by 7.6% per year from 2015 through 2023" are therefore not an adequate description of the time path.

**Figure 1:**
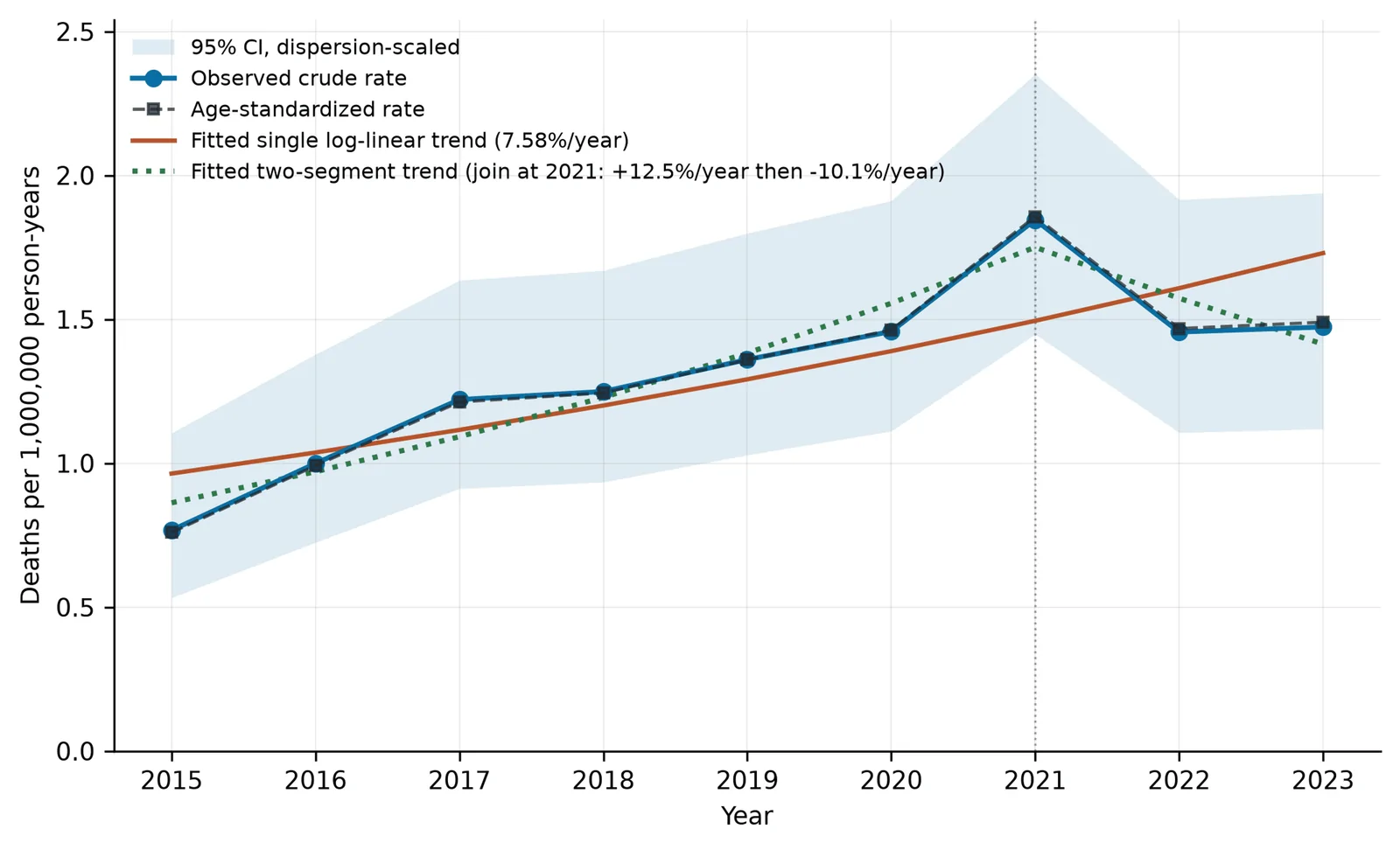
Annual mortality certified as diabetic ketoacidosis among Brazilian residents aged 0-19 years, 2015-2023 Observed crude rates with dispersion-scaled 95% confidence bands, directly age-standardized rates, the fitted single log-linear trend, and the fitted two-segment trend with a join in 2021. The single-slope model overpredicts the final two years

**Table 2:** Trend model estimates for mortality certified as diabetic ketoacidosis, Brazilian residents aged 0-19 years CI: confidence interval; RR: rate ratio Percent changes are annual. Intervals and p-values use the normal reference, as in the underlying generalized linear models. With nine annual observations, this reference is optimistic: referring the second segment to a t-distribution with the model's residual degrees of freedom moves its p-value from 0.02 to 0.058. The direction and magnitude of the slope change are the finding; its precision should not be overstated

Model	Estimate	95% CI	p	Dispersion
Single log-linear slope, 2015-2023	7.58% per year	3.66 to 11.65	<0.001	1.67
Segmented, 2015-2021	12.52% per year	9.22 to 15.91	<0.001	0.51
Segmented, 2021-2023	-10.12% per year	-17.80 to -1.73	0.02	0.51
Change of slope at 2021	-	-	<0.001	-
Age-adjusted single slope	7.87% per year	4.32 to 11.54	<0.001	1.35
Excluding 2021	6.64% per year	3.50 to 9.88	<0.001	0.99
Excluding 2020 and 2021	6.45% per year	3.19 to 9.81	<0.001	1.03
2020-2023 vs 2015-2019, crude	RR 1.40	1.11 to 1.75	0.004	2.37
2020-2023 vs 2015-2019, given trend	RR 1.03	0.68 to 1.55	0.91	1.93

Age standardization changed nothing material. Directly standardized rates were 0.759 per million in 2015 and 1.490 in 2023, against crude rates of 0.767 and 1.473, and the age-adjusted annual change was 7.87% (95% CI, 4.32%-11.54%) against the crude 7.58%. The fivefold gradient between age groups therefore does not distort the national trend, because the pediatric age structure changed little over nine years.

The pandemic period is not a step change

The pooled rate was 1.117 per million in 2015-2019 and 1.559 in 2020-2023, giving a crude RR of 1.40 (95% CI: 1.11-1.75; p = 0.004). Conditional on the continuous secular trend, however, the same contrast was 1.03 (95% CI: 0.68-1.55; p = 0.91), and adding the period indicator to the trend model did not improve fit (F = 0.01; df 1, 6; p = 0.91). The elevation of 2020-2023 relative to 2015-2019 is thus what the pre-existing trajectory already predicted, not an additional pandemic-era displacement. Excluding 2021 altogether left a trend of 6.64% per year (95% CI: 3.50%-9.88%), and excluding both 2020 and 2021 left 6.45% per year (95% CI: 3.19%-9.81%); notably, excluding 2021 also removed the extra-Poisson variation (dispersion fell from 1.67 to 0.99), identifying that single year as the source of the overdispersion.

Cause-of-death coding specificity and its contribution to the trend

Deaths attributed to diabetes at ages 0-19 years totaled 1,466 and moved from 137 in 2015 to 146 in 2023, an annual change of 2.59% (95% CI: 0.22%-5.01%). Over the same period the share of those deaths carrying a ketoacidosis fourth character rose from 35.0% to 58.2%, an annual relative increase in the share of 5.15% (95% CI: 2.78%-7.57%; p < 0.001), while diabetes deaths without a ketoacidosis fourth character did not increase (-1.85% per year; 95% CI: -4.55% to 0.92%; p = 0.19) (Table 3, Figure 2).

**Table 3:** Cause-of-death coding specificity: ketoacidosis as a fourth character among deaths attributed to diabetes, ages 0-19 years, 2015-2023 DKA: diabetic ketoacidosis All columns derive from the same SIM microdata extraction. "Mentioned anywhere" counts deaths with an E1x.1 code on any line of Part I or Part II of the certificate, irrespective of the underlying cause

Year	All diabetes deaths (E10-E14)	DKA deaths (E1x.1)	DKA share (%)	Diabetes deaths without E1x.1	DKA mentioned anywhere on certificate
2015	137	48	35.0	89	69
2016	157	62	39.5	95	90
2017	163	75	46.0	88	109
2018	166	76	45.8	90	104
2019	157	82	52.2	75	115
2020	177	87	49.2	90	135
2021	188	109	58.0	79	142
2022	175	85	48.6	90	122
2023	146	85	58.2	61	102
Total	1,466	709	48.4	757	988

Decomposing the 2015-to-2023 change in DKA deaths multiplicatively into a diabetes-mortality-volume component and a coding-share component attributes 88.9% of the rise to the change in coding share and 11.1% to the change in the number of diabetes deaths. By 2023, all-diabetes deaths had returned close to their 2015 level (146 vs 137) while the ketoacidosis share was at its maximum. The persistence of elevated certified DKA rates after the 2021 peak is therefore compatible with more specific certification of a roughly stable total of pediatric diabetes deaths.

The multiple-cause series supports the same reading. Deaths mentioning DKA anywhere on the certificate rose from 69 in 2015 to 102 in 2023, an increase of 47.8%, against 77.1% for the underlying-cause series; indexed to 2015, the two series track each other until 2021 and then diverge, with the multiple-cause count falling further in 2023 (Figure 2C).

**Figure 2:**
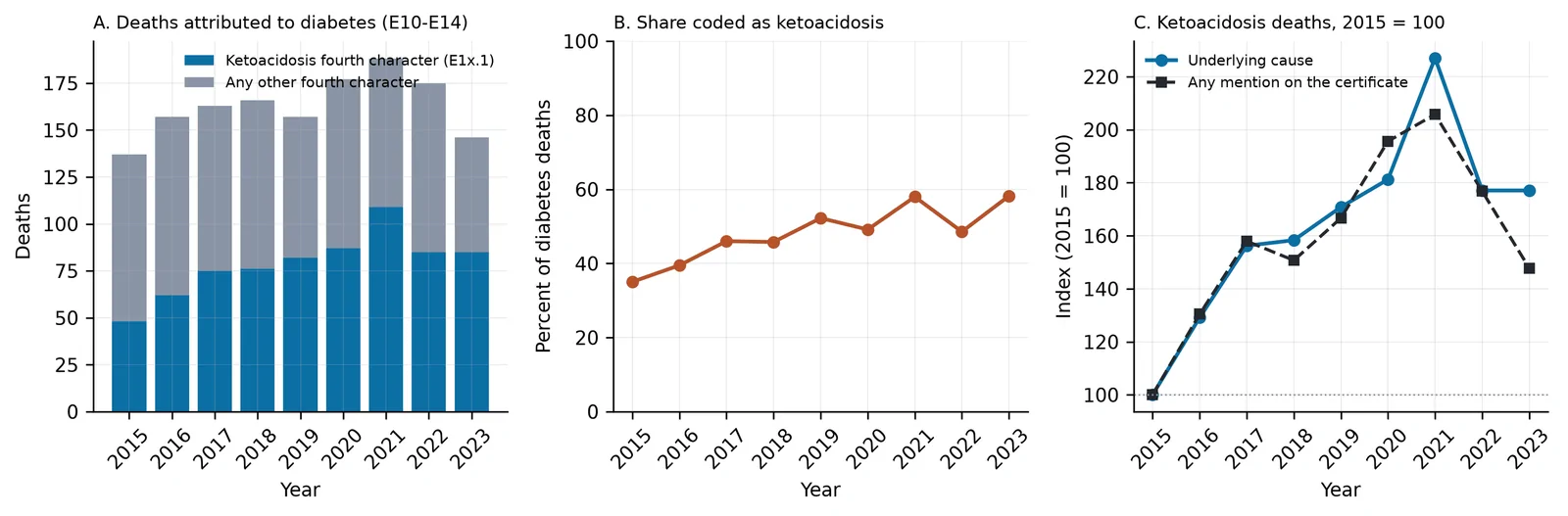
Cause-of-death coding specificity, ages 0-19 years, 2015-2023 (A) Annual deaths attributed to diabetes (E10-E14) separated by the presence of a ketoacidosis fourth character. (B) The share of those deaths coded as ketoacidosis. (C) Deaths certified as ketoacidosis by underlying cause and by any certificate mention, indexed to 2015. All series derive from the same microdata extraction

Sensitivity to case definition, age bounds, and period

The direction of the trend was robust to every alternative specification examined; its magnitude was not (Table 4, Figure 3).

**Table 4:** Annual percent change under alternative case definitions and age bounds APC: annual percent change; CI: confidence interval

Definition	Deaths	2015	2023	APC (%)	95% CI
E1x.1 underlying cause (primary)	709	48	85	7.58	3.66 to 11.65
E1x.1 or E1x.0 underlying cause	762	54	88	6.72	3.15 to 10.43
E1x.1 anywhere on the certificate	988	69	102	6.14	1.79 to 10.69
E1x.1 or E1x.0 anywhere on the certificate	1,041	75	104	5.28	1.15 to 9.57
All diabetes (E10-E14) underlying cause	1,466	137	146	2.59	0.22 to 5.01
Diabetes underlying cause, not E1x.1	757	89	61	-1.85	-4.55 to 0.92
E1x.1 as originally coded (CAUSABAS_O)	690	48	85	7.80	4.41 to 11.29
E1x.1 underlying, ages 0-14 only	323	20	46	8.77	3.28 to 14.55
E1x.1 underlying, ages 1-19 only	659	45	80	7.16	3.32 to 11.14
E1x.1 underlying, ages 15-19 only	386	28	39	6.61	-0.22 to 13.90

Adding diabetic coma (E1x.0) to the case definition added only 53 deaths across nine years and reduced the annual change modestly, from 7.58% to 6.72%. Widening to any certificate mention added 279 deaths and reduced it to 6.14%. Using the pre-investigation underlying-cause code gave 7.80%, showing that SIM investigation and recoding do not generate the trend. Restricting to ages 0-14 or 1-19 years, or to ages 15-19 years alone, left estimates between 6.61% and 8.77%, all with overlapping intervals.

**Figure 3:**
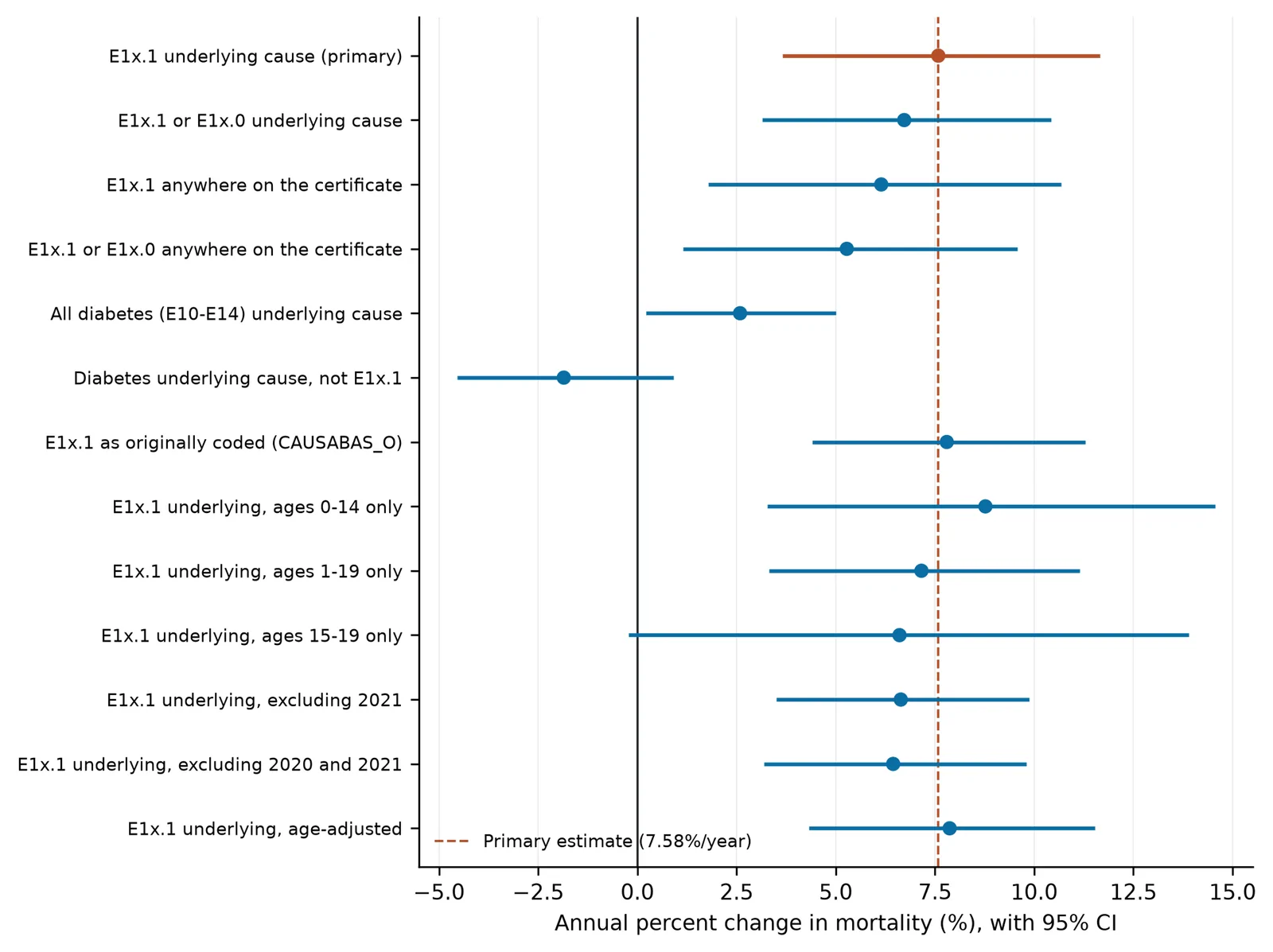
Annual percent change under each case definition and sensitivity analysis Point estimates with 95% confidence intervals; the dashed line marks the primary estimate

Age, sex, and regional variation

Adolescents aged 15-19 years accounted for 386 deaths and had the highest pooled rate, 2.654 per million person-years, 5.45 times the rate at ages 5-9 years (Table 5). Children aged 0-4 years had the second-highest rate, 1.163 per million.

**Table 5:** Mortality certified as diabetic ketoacidosis by age group, Brazil, 2015-2023 CI: confidence interval; RR: rate ratio Rate ratios come from a quasi-Poisson model fitted to year-by-age-group cells (dispersion: 2.11), so their intervals account for between-year variation

Age group, years	Deaths	Person-years	Rate per million (exact 95% CI)	RR vs 5-9 years (95% CI)
0-4	150	128,933,086	1.163 (0.985-1.365)	2.39 (1.56-3.66)
5-9	64	131,535,678	0.487 (0.375-0.621)	1.00 (reference)
10-14	109	136,058,468	0.801 (0.658-0.966)	1.65 (1.05-2.58)
15-19	386	145,455,409	2.654 (2.396-2.932)	5.45 (3.71-8.01)

Females accounted for 404 deaths (57.0%) and males for 305 (43.0%), giving rates of 1.524 and 1.102 per million person-years, respectively (RR: 1.38; 95% CI: 1.08-1.76; p = 0.009) (Table 6).

**Table 6:** Mortality certified as diabetic ketoacidosis by sex, Brazil, ages 0-19 years, 2015-2023 CI: confidence interval; RR: rate ratio Rate ratio from a quasi-Poisson model fitted to year-by-sex cells (dispersion: 2.68)

Sex	Deaths	Person-years	Rate per million (exact 95% CI)	RR (95% CI)
Male	305	276,873,849	1.102 (0.981-1.232)	1.00 (reference)
Female	404	265,108,792	1.524 (1.379-1.680)	1.38 (1.08-1.76)

The Northeast had the highest pooled rate and the South the lowest (RR: 1.74; 95% CI: 1.20-2.52; p = 0.003) (Table 7, Figure 4).

**Table 7:** Mortality certified as diabetic ketoacidosis by Brazilian macroregion, ages 0-19 years, 2015-2023 CI: confidence interval; RR: rate ratio Rate ratios from a quasi-Poisson model fitted to year-by-region cells (dispersion: 1.76). These are unadjusted pooled contrasts and are not estimates of independent risk or of care quality

Region	Deaths	Person-years	Rate per million (exact 95% CI)	RR vs South (95% CI)
North	72	58,576,586	1.229 (0.962-1.548)	1.41 (0.90-2.21)
Northeast	237	156,510,145	1.514 (1.328-1.720)	1.74 (1.20-2.52)
Southeast	279	210,847,301	1.323 (1.173-1.488)	1.52 (1.06-2.18)
South	63	72,357,957	0.871 (0.669-1.114)	1.00 (reference)
Central-West	58	43,690,652	1.328 (1.008-1.716)	1.52 (0.95-2.45)

**Figure 4:**
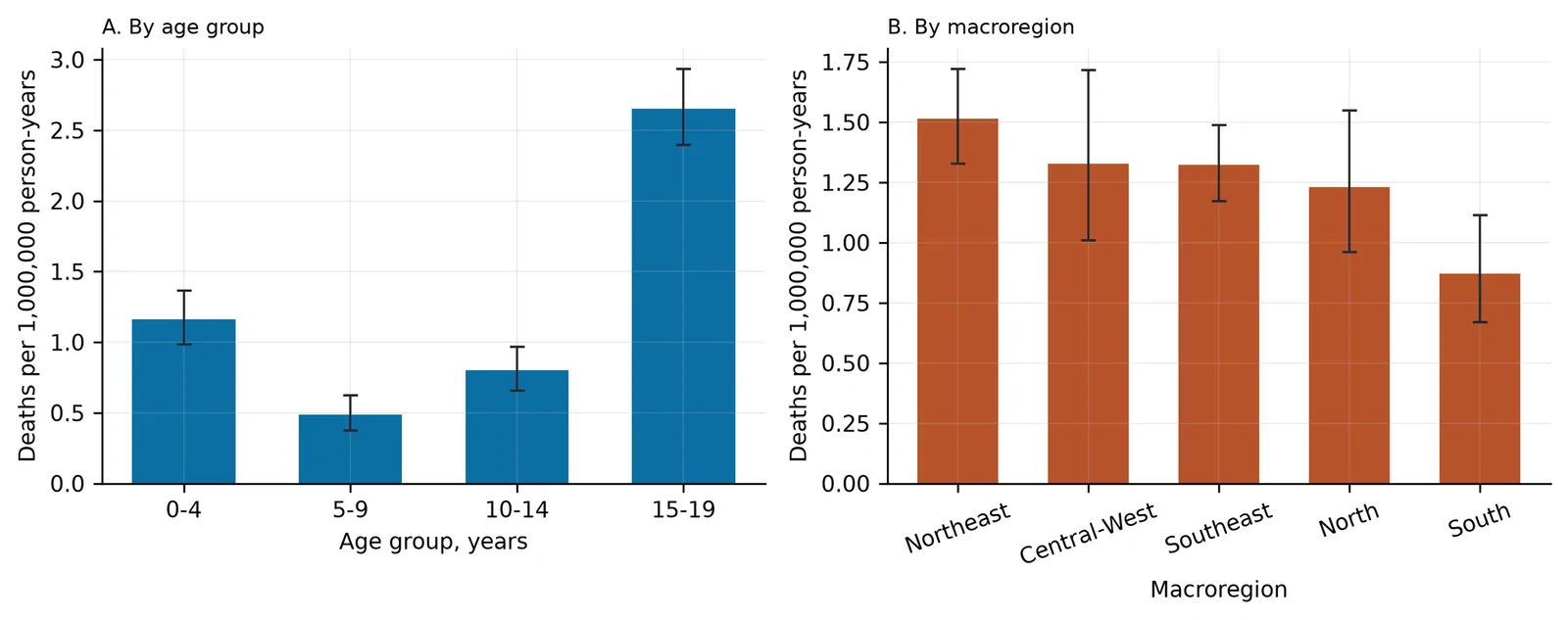
Pooled mortality rates certified as diabetic ketoacidosis by age group and Brazilian macroregion, 2015-2023 Rates are per one million person-years, with exact Poisson 95% confidence intervals

Death-certificate characteristics

Most deaths occurred in a hospital (642; 90.6%), followed by another health facility (55; 7.8%), home (8; 1.1%), public place (2; 0.3%), and other locations (2; 0.3%). Race/color was recorded as Brown/mixed in 352 deaths (49.6%), White in 272 (38.4%), Black in 45 (6.3%), Indigenous in nine (1.3%), Asian in five (0.7%), and unknown in 26 (3.7%).

Unspecified diabetes with ketoacidosis (E14.1) accounted for 420 deaths (59.2%), type 1 diabetes with ketoacidosis (E10.1) for 244 (34.4%), type 2 diabetes (E11.1) for 32 (4.5%), other specified diabetes (E13.1) for nine (1.3%), and malnutrition-related diabetes (E12.1) for four (0.6%) (Table 8). The high proportion of E14.1 precluded analysis by diabetes type.

**Table 8:** Death-certificate characteristics of deaths certified as diabetic ketoacidosis, Brazil, ages 0-19 years, 2015-2023 ICD-10: International Classification of Diseases, 10th Revision Percentages describe certified deaths and are not population-specific mortality risks. Race/color is as recorded on the death certificate; no race-specific rates are estimated

Variable	Category	Deaths	%
Race/color	Brown/mixed	352	49.6
Race/color	White	272	38.4
Race/color	Black	45	6.3
Race/color	Unknown	26	3.7
Race/color	Indigenous	9	1.3
Race/color	Asian	5	0.7
Place of death	Hospital	642	90.6
Place of death	Other health facility	55	7.8
Place of death	Home	8	1.1
Place of death	Public place	2	0.3
Place of death	Other	2	0.3
ICD-10 code	E14.1 (unspecified diabetes)	420	59.2
ICD-10 code	E10.1 (type 1 diabetes)	244	34.4
ICD-10 code	E11.1 (type 2 diabetes)	32	4.5
ICD-10 code	E13.1 (other specified)	9	1.3
ICD-10 code	E12.1 (malnutrition-related)	4	0.6

## Discussion

In this national study, mortality certified as DKA among Brazilian children and adolescents rose from 2015 to a peak in 2021 and then declined, remaining in 2022-2023 above every pre-2020 annual rate. Three findings qualify that description and are, in our view, the substantive contribution of the study. First, the time path is not a constant annual increase: a segmented model gives +12.5% per year to 2021 and -10.1% per year thereafter, and the conventional single-slope summary overpredicts the end of the series. Second, most of the measured rise is attributable to increasing specificity of fourth-character coding rather than to a proportional increase in diabetes deaths: the decomposition assigns 88.9% of the 2015-to-2023 change to the coding share. Third, the elevation of 2020-2023 over 2015-2019, although real as a period contrast, is entirely explained by the secular trend once that trend is modeled, and therefore provides no evidence of a pandemic-specific step change.

What the data support, and what they do not

These files identify deaths whose certificate carried a particular code. They support statements about certified underlying-cause mortality, about its distribution by age, sex, and region, and about how the fourth character was applied over time. They do not establish that fatal ketoacidosis itself became more common. Distinguishing a true increase in fatal DKA from more specific labeling of a stable set of diabetes deaths would require linkage to clinical, laboratory, or registry data, which SIM does not provide. We therefore frame the persistence of elevated certified rates as a surveillance signal that warrants clinical investigation, not as demonstrated evidence that preventable deaths became more frequent.

The comparison with all pediatric diabetes deaths is what makes this distinction measurable rather than speculative. Total diabetes mortality at ages 0-19 years changed little across the period, ending in 2023 at 146 deaths against 137 in 2015, while the proportion carrying a ketoacidosis fourth character rose from about one third to nearly three fifths, and diabetes deaths without that character declined. Multiple-cause data point the same way: mentions of ketoacidosis anywhere on the certificate rose by 48% between 2015 and 2023 against 77% for the underlying-cause series, and the two diverge after 2021. Multiple-cause analysis is a standard corrective to shifts in underlying-cause assignment [20], and here it suggests that part of what the primary series records is a migration of the same events into a more specific code rather than an increase in the events themselves.

Comparison with previous work

The result extends earlier Brazilian evidence. Negrato et al. found DKA at diagnosis in 42.3% of a multicenter type 1 diabetes sample, with age, economic status, and region associated with presentation [7]. A Curitiba cohort also reported frequent DKA at diagnosis and highlighted missed opportunities for earlier recognition [8]. Merino et al. evaluated broad diabetes deaths through 2015 and found declining overall pediatric diabetes mortality but an exception in those aged 15-19 years [9]. Our four-character definition isolates ketoacidosis rather than all diabetes deaths; the present analysis shows that this gain in clinical specificity comes at the cost of sensitivity to coding practice, and that the two must be reported together.

International reports of more frequent pediatric DKA at diabetes onset during the COVID-19 period [11-13] are consistent in direction with our 2021 peak. However, our increase began well before 2020; the series contains only nine points, mortality data do not identify SARS-CoV-2 infection or healthcare disruption, and the period contrast is not distinguishable from the pre-existing trend. The peak in 2021 and the subsequent decline are compatible with several explanations, including a genuine transient rise, the maturation of a coding shift, and chance in a series with fewer than 110 deaths in its largest year. This study does not estimate a causal pandemic effect.

Demographic and regional variation

The highest rate at ages 15-19 years is clinically plausible but should not be confused with the frequency of DKA at first diabetes diagnosis. International studies often find high DKA-at-onset risk in preschool children, while adolescents face hazards from established diabetes, including insulin omission, treatment fatigue, mental-health conditions, stigma, substance use, unstable transitions between pediatric and adult care, and loss of family supervision [1,3,5]. SIM cannot distinguish new-onset from recurrent DKA or identify insulin access and psychosocial circumstances. The elevated rate at ages 0-4 years remains important because diagnostic delay can progress rapidly in young children.

The higher female rate requires further investigation. Multinational data have sometimes found female sex associated with DKA at diagnosis in specific countries, but patterns are inconsistent and do not establish greater biological susceptibility [5]. Sex differences in fatal DKA could arise from diabetes type, treatment behavior, eating disorders, healthcare-seeking, deprivation, or coding.

The Northeast-to-South gradient may reflect differences in timely diagnosis, specialist distribution, continuous insulin and supply access, transport, emergency protocols, intensive care, and social vulnerability. It may equally reflect variation in death registration, in the completeness of certification, and in the propensity to assign a specific fourth character. National SIM completeness is high, but residual subnational underregistration remains [15]. These are unadjusted pooled contrasts; they are surveillance indicators, not measures of care quality, and should not be read as independent risk effects.

The predominance of E14.1 is itself a surveillance finding. Diabetes type was unspecified in 59.2% of fatal DKA records, even though type 1 diabetes is generally expected to dominate pediatric DKA. This prevents valid attribution of trends to type 1 or type 2 diabetes and may obscure emerging phenotypes. Improving death-certificate documentation and linking SIM to hospital, laboratory, pharmacy, and diabetes-registry data would materially strengthen prevention planning.

Strengths and limitations

The study uses final national resident-death files rather than a hospital sample; applies a four-character ICD-10 definition that is more specific than the three-character diabetes grouping used in earlier Brazilian work; uses official age-, sex-, and region-specific denominators from a single documented population vintage; derives both the DKA series and the all-diabetes comparison from the same microdata extraction, which reproduces the published TabNet counts exactly; and reports the case definition, sensitivity analyses, and dispersion scaling explicitly enough to be checked. Extraction and analysis scripts, a data dictionary of SIM fields and filters, the eligible-case file, denominator tables, and all outputs are deposited in a public repository (DOI 10.5281/zenodo.22802945).

Several limitations are material to interpretation. First, and most important, this is a study of certified cause of death. Death certificates are vulnerable to diagnostic and coding error; biochemical DKA criteria could not be validated; and the analyses above show that coding specificity accounts for most of the measured change. The primary estimate should not be read as the rate of change of fatal ketoacidosis.

Second, the operational definition is narrower than the clinical entity. In ICD-10, E1x.1 is ketoacidosis without mention of coma while E1x.0 is diabetic coma with or without ketoacidosis [16]; fatal pediatric DKA frequently involves coma or cerebral injury, so the primary definition can exclude the most severe presentations. In these data, that exclusion was numerically small (53 deaths over nine years), and adding E1x.0 reduced the annual change from 7.58% to 6.72%, but the conceptual mismatch between code and clinical event remains.

Third, restriction to the underlying cause misses DKA recorded only as a contributing condition; the multiple-cause analysis addresses this and yields a smaller annual change.

Fourth, the denominator is the general population aged 0-19 years, not children with diabetes. A rising incidence of type 1 diabetes would raise the population rate without any change in the risk faced by an individual child with diabetes. These data cannot separate incidence, case fatality, registration completeness, and coding practice.

Fifth, a nine-point ecological series cannot establish individual risk factors or causality, including a pandemic effect, and the location of the 2021 join is descriptive: with this many points, we did not apply the permutation-based selection used for longer series [17], and the knot should not be treated as a precisely estimated changepoint. For the same reason, the normal reference used for all intervals is optimistic; the declining second segment, for example, has p = 0.02 against a normal reference and p = 0.058 against a t reference on the model's residual degrees of freedom. We report the direction and approximate magnitude of the change in slope, not a precise date or rate of decline.

Sixth, we did not estimate in-hospital case fatality. The Hospital Information System (SIH) records admissions and could support an approximate hospital-based denominator, but SIH is an administrative billing system, DKA admissions are subject to their own coding variation, and SIH records cannot be linked to SIM deaths in the public files. Such an analysis is a distinct study with distinct biases, and we did not attempt it here.

Seventh, race/color percentages describe certified deaths and lack compatible joint denominators; they are not risks. Regional comparisons may reflect ascertainment as well as occurrence. Ill-defined causes accounted for 3.4% of pediatric deaths and may conceal some DKA deaths in every year.

Finally, age standardization and age adjustment changed the trend estimate only trivially, so age structure is not a competing explanation for the national trajectory; we report it because the five-fold gradient across age groups makes the question a reasonable one.

Implications

The following are clinically orthodox measures whose justification rests on the established preventability of DKA and on the existence of these deaths,not on findings of this study. These data contain no information on diagnostic delay, insulin supply, sick-day practice, or hospital capability, and a nine-year ecological series cannot evaluate any intervention.

For children not yet diagnosed, sustained public and clinician education about polyuria, polydipsia, nocturia, weight loss, fatigue, vomiting, and abnormal breathing can reduce diagnostic delay; campaign reviews report reductions in DKA when interventions are sustained and target both the community and frontline professionals [6,21]. For children with established diabetes, reliable insulin and ketone-testing access, written sick-day plans, 24-hour advice, psychosocial screening, school support, and structured transition programs are central. At hospital level, standardized pathways, early recognition of cerebral injury, and experienced multidisciplinary care remain essential [1,4].

The findings of this study bear on a different target: the quality of the surveillance instrument itself. Improving the specificity and consistency of pediatric diabetes death certification, reducing the 59.2% share of unspecified-type coding, and establishing linkage between mortality records and clinical episodes would allow the question this study could not answer, whether fatal ketoacidosis is becoming more frequent, to be answered directly.

## Conclusions

Among Brazilian residents aged 0-19 years, mortality certified as DKA (ICD-10 E1x.1, underlying cause) rose from 2015 to a peak in 2021, declined thereafter, and was higher in 2020-2023 than in 2015-2019. A single annual rate of change describes this path poorly, and the period contrast is fully accounted for by the secular trend rather than by a pandemic-era step change. Most of the increase in certified DKA deaths is consistent with more specific fourth-character coding rather than with a proportional rise in deaths attributed to diabetes, which changed little over the period. Rates were highest among adolescents aged 15-19 years, higher in females than males, and higher in the Northeast than in the South, as unadjusted pooled contrasts. Predominance of unspecified diabetes (E14.1) precludes etiologic interpretation by diabetes type.

Causal inference about the pandemic, about clinical mechanisms, and about the expected effect of preventive interventions is not warranted by this design. Future research should validate death-certificate coding against clinical records and link mortality data to hospital, pharmacy, and registry sources, so that diagnostic delay, recurrent DKA, treatment barriers, and health-system factors can be distinguished from changes in how deaths are classified.

## References

[1] Glaser N, Fritsch M, Priyambada L (2022). ISPAD clinical practice consensus guidelines 2022: diabetic ketoacidosis and hyperglycemic hyperosmolar state. Pediatr Diabetes.

[2] Libman I, Haynes A, Lyons S (2022). ISPAD Clinical Practice Consensus Guidelines 2022: definition, epidemiology, and classification of diabetes in children and adolescents. Pediatr Diabetes.

[3] Usher-Smith JA, Thompson MJ, Sharp SJ, Walter FM (2011). Factors associated with the presence of diabetic ketoacidosis at diagnosis of diabetes in children and young adults: a systematic review. BMJ.

[4] Azova S, Rapaport R, Wolfsdorf J (2021). Brain injury in children with diabetic ketoacidosis: review of the literature and a proposed pathophysiologic pathway for the development of cerebral edema. Pediatr Diabetes.

[5] Cherubini V, Grimsmann JM, Åkesson K (2020). Temporal trends in diabetic ketoacidosis at diagnosis of paediatric type 1 diabetes between 2006 and 2016: results from 13 countries in three continents. Diabetologia.

[6] Minerba E, Maines E, Quaglia N, Fedi L, Fanti S, Fierro A, Mozzillo E (2024). Diabetes awareness campaigns to prevent ketoacidosis at the diagnosis of type 1 diabetes: efficacy on multiple outcomes and predictors of success: a systematic review. J Pers Med.

[7] Negrato CA, Cobas RA, Gomes MB (2012). Temporal changes in the diagnosis of type 1 diabetes by diabetic ketoacidosis in Brazil: a nationwide survey. Diabet Med.

[8] Souza LC, Kraemer GC, Koliski A, Carreiro JE, Cat MN, Lacerda L, França SN (2020). Diabetic ketoacidosis as the initial presentation of type 1 diabetes in children and adolescents: epidemiological study in Southern Brazil. Rev Paul Pediatr.

[9] Merino MF, Oliveira RR, Silva PL, Carvalho MD, Pelloso SM, Higarashi IH (2019). Hospitalization and mortality by diabetes mellitus in children: analysis of temporal series. Rev Bras Enferm.

[10] Costa LF, Sampaio TL, Moura L, Rosa RD, Iser BP (2024). Time trend and costs of hospitalizations with diabetes mellitus as main diagnosis in the Brazilian National Health System, 2011 to 2019. Epidemiol Serv Saude.

[11] Birkebaek NH, Kamrath C, Grimsmann JM (2022). Impact of the COVID-19 pandemic on long-term trends in the prevalence of diabetic ketoacidosis at diagnosis of paediatric type 1 diabetes: an international multicentre study based on data from 13 national diabetes registries. Lancet Diabetes Endocrinol.

[12] D'Souza D, Empringham J, Pechlivanoglou P, Uleryk EM, Cohen E, Shulman R (2023). Incidence of diabetes in children and adolescents during the COVID-19 pandemic: a systematic review and meta-analysis. JAMA Netw Open.

[13] Elgenidy A, Awad AK, Saad K (2023). Incidence of diabetic ketoacidosis during COVID-19 pandemic: a meta-analysis of 124,597 children with diabetes. Pediatr Res.

[14] Saldanha RF, Bastos RR, Barcellos C (2019). Microdatasus: a package for downloading and preprocessing microdata from Brazilian Health Informatics Department (DATASUS) (Article in Portuguese). Cad Saude Publica.

[15] Costa LF, de Mesquita Silva Montenegro M, Rabello Neto DL, de Oliveira AT, Trindade JE, Adair T, Marinho MF (2020). Estimating completeness of national and subnational death reporting in Brazil: application of record linkage methods. Popul Health Metr.

[16] (2026). International Statistical Classification of Diseases and Related Health Problems, 10th Revision (ICD-10), 2019 version. https://icd.who.int/browse10/2019/en.

[17] Kim HJ, Fay MP, Feuer EJ, Midthune DN (2000). Permutation tests for joinpoint regression with applications to cancer rates. Stat Med.

[18] Fay MP, Feuer EJ (1997). Confidence intervals for directly standardized rates: a method based on the gamma distribution. Stat Med.

[19] Resolução nº 510, de 7 de abril de 2016. (2016 (2026). Resolução nº 510, de 7 de Abril de 2016. https://www.gov.br/conselho-nacional-de-saude/pt-br/atos-normativos/resolucoes/2016/resolucao-no-510.pdf.

[20] Redelings MD, Sorvillo F, Simon P (2006). A comparison of underlying cause and multiple causes of death: US vital statistics, 2000-2001. Epidemiology.

[21] Cherubini V, Marino M, Carle F, Zagaroli L, Bowers R, Gesuita R (2021). Effectiveness of ketoacidosis prevention campaigns at diagnosis of type 1 diabetes in children: a systematic review and meta-analysis. Diabetes Res Clin Pract.

